# Carrageenans-Sulfated Polysaccharides from Red Seaweeds as Matrices for the Inclusion of Echinochrome

**DOI:** 10.3390/md15110337

**Published:** 2017-11-01

**Authors:** Irina M. Yermak, Natalia P. Mischchenko, Viktoria N. Davydova, Valery P. Glazunov, Daria V. Tarbeeva, Anna O. Kravchenko, Evgeniya A. Pimenova, Irina V. Sorokina

**Affiliations:** 1 G.B. Elyakov Pacific Institute of Bioorganic Chemistry, Far-Eastern Branch of the Russian Academy of Sciences, 100 Let Vladivostoku Prosp., 159, 690022 Vladivostok, Russia; mischenkonp@mail.ru (N.P.M.); viktoria@piboc.dvo.ru (V.N.D.); glazunov@piboc.dvo.ru (V.P.G.); tarbeeva1988@gmail.com (D.V.T.); kravchenko_25.89@mail.ru (A.O.K.); 2 National Scientific Center of Marine Biology, Far-Eastern Branch of the Russian Academy of Sciences, Palchevskogo, 17, 690041 Vladivostok, Russia; ea_pimenova@mail.ru; 3 N.N. Vorozhtsov Novosibirsk Institute of Organic Chemistry, Siberian Branch of the Russian Academy of Sciences, Lavrentjev Ave. 9, 630090 Novosibirsk, Russia; sorokina@nioch.nsc.ru

**Keywords:** carrageenan, echinochrome, spectra, release, gastroprotective activity

## Abstract

The possibility of using different types of carrageenans (CRG) as matrixes for incorporating of echinochrome A (Ech) was investigated. Ech interacts with carrageenans and is incorporated into the macromolecular structure of the polysaccharide. The inclusion of Ech in carrageenan matrices decreased its oxidative degradation and improved its solubility. The changing in the charge and morphology of CRGs during binding with Ech was observed. The rate of Ech release from CRG matrices depended on the structure of the used polysaccharide and the presence of specific ions. The gastroprotective effect of CRG/Ech complexes was investigated on the model of stomach ulcers induced by indomethacin in rats. Complexes of CRG/Ech exhibited significant gastroprotective activity that exceeded the activity of the reference drug Phosphalugel. The gastroprotective effect of the complexes can be associated with their protective layer on the surface of the mucous membrane of a stomach.

## 1. Introduction

Natural polymers possess unique characteristics as they are inert, safe, nontoxic, cheap, eco-friendly, and abundantly available in nature. Unlike synthetic polymers, natural polysaccharides of various chemical structures exhibit important properties, such as biocompatibility, biodegradability, adhesiveness, the ability to form hydrogels [1]. Thus, they have attracted the interest of researchers in the biomedical and biopharmaceutical fields [2,3]. The red algae are mostly composed of sulphated galactans such as agars or carrageenans which are not found in land plants, and have a wide application in practice. Carrageenans (CRGs) are sulfated linear galactans with the basic structural units of carrabiose disaccharides, which consist of alternating β-1,3- and α-1,4-linked galactose residues [4]. The variations in its basic structure are determined by the occurrence of 3,6-anhydrogalactose and the location and number of sulfate groups in the linked galactose residues.

The three most industrially exploited types, namely, kappa-, iota- and lambda-CRGs, are distinguished by the presence of one, two and three ester sulfate groups per repeating disaccharide unit, respectively [4]. Native CRGs always represent complex hybrid structures or, generally, a mixture of galactans composed of different carrabiose types, the proportions and structures of which vary with the species and ecophysiological and developmental conditions [5,6]. CRGs are widely utilized in the food industry due to their excellent physical functional properties, such as their thickening, gelling and stabilizing abilities [6]. Intensive studies have shown that CRGs can be regarded for applications besides foodstuff ingredients due to their wide spectrum of biological and physiological activities, such as their antiviral [7], anticoagulant [8], antitumor [9], and immunomodulatory properties [10,11]. Previously, we have shown the possibility of the potential use of carrageenan as agents for treating endotoxemic complications from Gram-negative infections, such as sepsis and endotoxic shock [12,13].

At present, CRGs has already been included in the United States Pharmacopeia 35-National Formulary 30S1 (USP35-NF30S1), British Pharmacopoeia 2012 (BP2012) and European Pharmacopoeia 7.0 (EP7.0), implying that CRGs may have a promising future as a pharmaceutical excipient [14]. Due to the diverse physicochemical properties of these polysaccharides, such as their high molecular weights, high viscosities and ability to form gels [15], CRGs are used by pharmaceutical researchers to improve drug formulations [16], prolong drug release [17] and create pH-temperature-sensitive delivery systems that can adjust to their physiological environments [18]. Complexation with CRGs facilitates the passage of drugs into aqueous suspensions and imparts them with sustained action [19]. CRGs, in addition to its good gelation properties, exhibits adhesive characteristics due in part to its molecular interactions with mucosal tissues [20]. 

The oral route of drug administration remains the most preferable method among all others in terms of patient tolerance. Carrageenan-based formulations could enable orally administered drugs to be released with a zero-order profile or within a significantly prolonged period of time. The use of CRGs as an excipient for oral delivery depends mostly on their degree of sulfation and physicochemical properties, such as their water solubility and jellification capability, which affect their mechanical properties and dissolution rate. These factors may affect the release of the active substance, prolonging or accelerating its release [21]. Due to its high ability to adsorb water, CRGs can improve drug dissolution and thus increase the oral bioavailability of poorly water-soluble drugs [20]. 

Echinochrome A (Ech, 6-ethyl-2,3,5,7,8-pentahydroxy-1,4-naphthoquinone) is the most common red pigment in sea urchins [22]. Ech, a water-insoluble compound, is the active substance (P N002362/01) of the drug Histochrome, which is registered in the Russian Federation as a solution for injections. Histochrome is used for the treatment of ocular diseases, such as intraocular haemorrhages, diabetic retinopathy, dystrophies, central retinal vein thrombosis, and post-traumatic haemorrhages [23,24]. Histochrome is also used for preventing reperfusion damage during myocardial infarction [25]. This medication exhibits high antioxidant activity, activated energy production and gene expression for the mitochondria in cardiomyocytes and increases oxidative phosphorylation in the mitochondria content in animal skeletal muscles [26], increasing the tolerance of animals to physical loads [27]. After the ampoules are opened, the Histochrome solution easily undergoes oxidation due to the oxygen in air. Therefore, developing new dosage forms of Histochrome that can increase the Ech solubility, protect its hydroxyl groups from oxidation, and reinforce its pharmacological properties is an important task. 

The inclusion of Ech in complexes with polysaccharides has been proposed to reduce its oxidative degradation. New polysaccharide-based matrices may also be beneficial for oral administration and the prolonged action of Histochrome. The aim of this work is to study the possibility of using of different types of CRGs as matrices that include Ech to protect and improve the drug dissolution as well as for modifying its pharmacological properties.

## 2. Results

### 2.1. Types of CRGs and Ech

The CRGs were extracted from red seaweed and separated using 4% KCl into KCl-insoluble and KCl-soluble fractions. The structures of polysaccharides were studied by ^13^C-NMR and FTIR-spectroscopy. The obtained spectra have been compared with the spectra of polysaccharides isolated by us earlier from these species of algae [28,29,30]. The identity of the spectra indicated that the KCl-insoluble fraction from *Chondrus armatus* (Rhodophyta, family Gigartinaceae) was κ-CRG and KCl-soluble fractions—λ-CRG [28]. According to data obtained by ^13^C-NMR and FT IR-spectroscopy, KCl-insoluble polysaccharides from *Tichocarpus crinitus* (Rhodophyta, family Tichocarpaceae) and *Ahnfeltiopsis flabelliformis* (Rhodophyta, family Phyllophoraceae) have hybrid structures. Based on the analysis of spectra compared with data obtained previously these polysaccharides have been identified as κ/β-CRG from *T. crinitus* [29] and ι/β-CRG from *A. flabelliformis* [30]. The chemical structures of the disaccharide-repeating units of the CRGs and viscosimetric molecular weights of CRGs, calculated by the Mark–Houwink, are listed in Table 1.

The CRGs differed from each other by their degree of sulfation, the positions of their sulfate groups and by the presence of 3,6-anhydrocalactose residues and hybrid structures. Molecular weights of CRGs were more than 100 kDa. 

The standardized substance Echinochrome (pentahydroxyethylnaphthoquinone, Ech), registration number in the Russian Federation is P N002362/01 (Russian State Register of Drugs (as of 5 December 2016) Part 2), was obtained in G.B. Elyakov Pacific Institute of Bioorganic Chemistry, Vladivostok in powder form. We used an ethanolic solution of Ech in concentration 10 mg/mL as a stock solution.

### 2.2. The Solubility of Ech and the Protective Effect of CRG on the Ech Oxidation by Air Oxygen 

To study the solubility of Ech in the present polysaccharides, water solutions with various types of CRG were used: κ-CRG, κ/β-CRG, ι/κ-CRG and λ-CRG. The solutions were prepared as described above with a CRG/Ech weight ratio of 5:1 and the absorption of Ech was recorded at 472 nm. The absorption of Ech in the CRG water solutions was constant over 24 h, indicating the solubility of Ech (approximately 0.1 mg × mL^−1^) in the water solutions of all types of CRGs while absorption of Ech in water alone decreased more than twice.

To evaluate the protective effect of CGR on the oxidation of Ech the contents Ech in the λ-CRG and κ-CRG solutions (pH 5.85) were determined by measuring the absorption values at 472 nm. The same absorption measurements were conducted using a 0.01 M phosphate buffer (pH 5.9) solution of Ech. According to obtained results the Ech in the CGR solutions (pH 5.85) oxidized much more slowly than in the phosphate buffer solution. Furthermore, over 2 days, 63% and 92% of the Ech was preserved in solutions of κ- and λ-CGR under light exposure, whereas in a phosphate buffer solution, 80% of the Ech was oxidized. Likewise, after 72 h, the content of the native Ech in the λ-CRG solution was one and a half times higher in comparison to its content in the κ-CRG solution.

**Figure 1:**
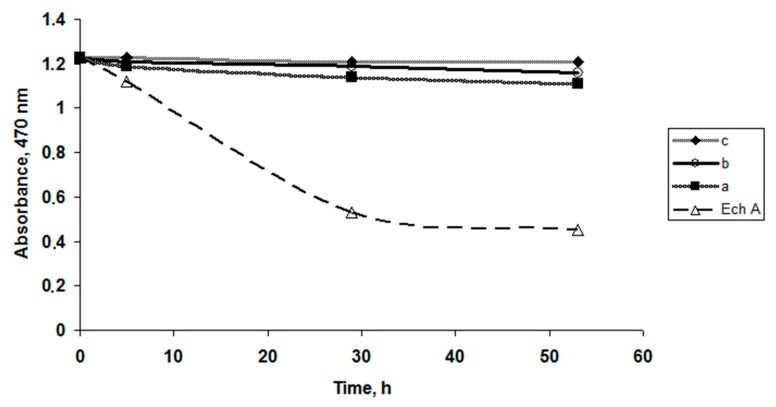
Stability of Ech in aqueous solutions and in solutions with κ-CGR. The ratios of CGR and Ech in the solutions are a—5:1; b—10:1; and c—50:1.

To evaluate the content of κ-CGR on the stability or oxidation of the Ech solutions, various CGR concentrations were used. The dependence of the absorption values of Ech as a function of time, shown in Figure 1, indicates that the Ech concentration (C = 0.1 mg × mL^−1^) in the CGR solutions remained constant at high polysaccharide concentrations compared to the water solutions. A slight change in the Ech concentration was observed in solutions with a small concentration of CGR: 0.5 mg × mL^−1^ and 1 mg × mL^−1^ (Figure 1a,b).

In solutions with a high concentration of CRG (5 mg × mL^−1^), the concentration of Ech did not change, which indicates that the Ech in the CGR solutions was not oxidized (Figure 1c). 

### 2.3. Differential Absorption Spectra of the CRG and Ech 

**Figure 2:**
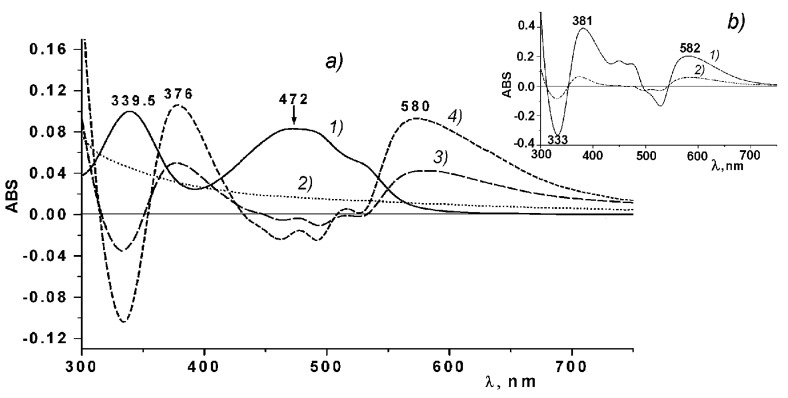
(**a**) Absorption spectra in water-alcohol solutions: Ech—line a1; ι/κ-CRG (C = 2 mg × mL^−1^)—line a2; differential spectra of CRG/Ech 8:1—line a3; and 25:1—line a4. (**b**)—Differential spectra of Ech in the phosphate buffer (pH 5.9)—line b1; Ech + lambda-carrageenan—line b2.

To study the possibility of binding between Ech and CRG, differential spectra measurements of Ech were carried out. Figure 2 shows the differential spectra and absorption spectra of Ech and ι/κ-CGR. As seen from Figure 2a, two absorption bands characteristic of the Ech at 339 nm and 472 nm can be observed in the absorption spectrum of Ech. In the differential spectra of the Ech in solutions of CGR, two positive bands are present at 376 and 580 nm, which may be indicative of the binding between Ech and CRG. Analogous differential spectra of Ech were obtained also for the other types of CGR. 

The differential spectra of Ech qualitatively coincide with the differential spectra of Ech in the 0.01 M phosphate buffer (pH 5.9) in regard to the number of bands and their locations (Figure 2b).

### 2.4. Morphology of the CRG/Ech Complexes 

**Figure 3:**
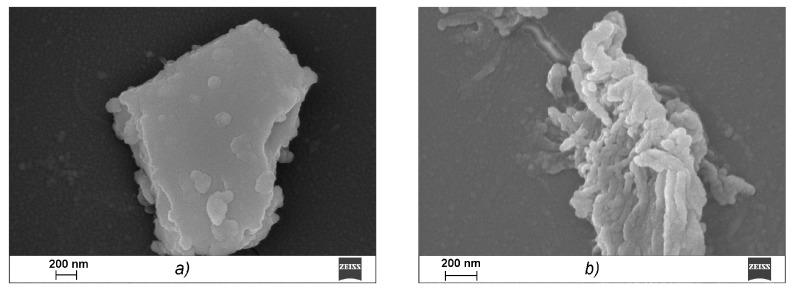

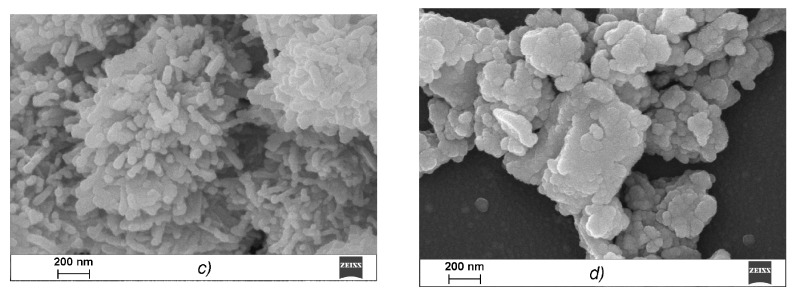
Scanning electron microscopy images of κ-CRG (**a**), C = 0.5 mg × mL^−1^, Ech (**b**) C = 0.1 mg × mL^−1^ and their mixture ((**c**) ratio CRG/Ech 5:1 *w*/*w*) and (**d**) ratio CRG/Ech 10:1 *w*/*w*) in water. Mag. = 50.87 KX, EHT = 10.00 kV.

A scanning electron microscope (SEM) was used to study the microstructures of the CRG/Ech complexes. κ-CGR, as seen from the data presented in Figure 3a, forms a plate laminated structure with a fairly smooth surface, which is characteristic of solutions of CGR in a concentration range of 0.1 to 5 mg × mL^−1^. Ech is presented in the images in the form of elongated cylinders that were collected from small bead-like structures, in contrast to CRG (Figure 3b). After incubating CRG with Ech, none of the large plate laminated structures remained, and the image shows a layered formation composed of particles of various shapes and sizes (Figure 3c). With an increase in the CRG concentration in the mixture (CRG/Ech—10:1), more densely packed sponge-like structures formed from the fractured beads (Figure 3d). Figure 3c,d presents a loose sponge-like structure in which interactions clearly occurred throughout the carrageenan network, not only on the surface, which resulted in this sponge-like format.

### 2.5. Electrophoretic Properties of the CRG/Ech Complexes

**Figure 4:**
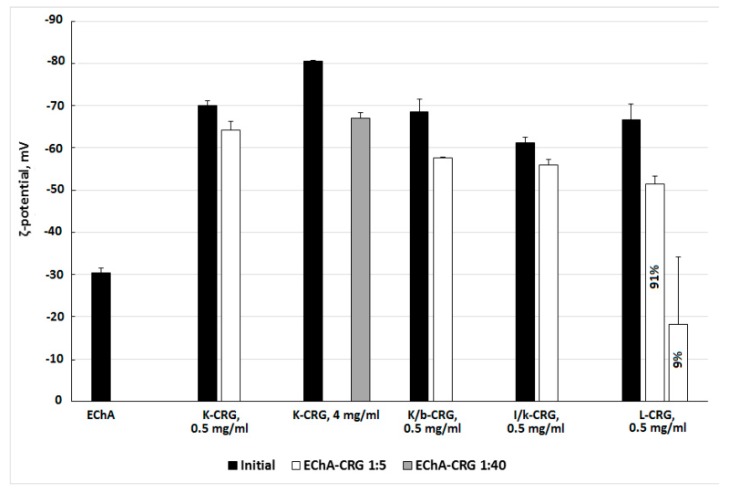
ζ-potentials of CRGs and its complexes with Ech.

The formation of complexes of different types of CRG with Ech was monitored using electrokinetic measurements. The results of the ζ-potential measurements of CRG and its complexes with Ech are presented in Figure 4.

The particle size distribution in the CRG solutions, characterized using dynamic light scattering, revealed a distribution with a high polydispersity typical for linear polysaccharide. The measurements of the zeta (ζ)-potentials of CRG showed that under the experimental conditions (C_CRG_ = 0.5 mg × mL^−1^), the CRG particles were negatively charged. The surface charges of the different types of CGR varied from −70 mV for κ-CRG to −61 mV for ι/κ-CRG. Ech formed large particles in solution with sizes from 171 to 652 nm and a negative charge of −30 mV. The addition of Ech to solutions of CRG resulted in the formation of a mixture that changed the charge of CRG (Figure 4). In the case of λ-CRG-Ech mixture the formation of two populations of particles varying in ζ-potential value was observed (Figure 4). 

### 2.6. The Release of Ech from Solutions with CRG 

**Figure 5:**
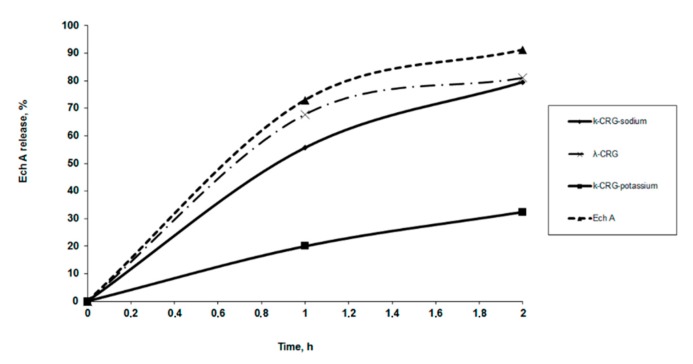
Ech release kinetics from solutions with κ- (κ-sodium and κ-potassium salt) and λ-CRG (CRG/Ech 10:1), in the medium simulating human stomach conditions. Concentration of Ech 0.1 mg × mL^−1^.

The Ech release from solutions with CRG in vitro was measured using a dialysis method with a membrane that simulated human stomach conditions. The release profiles of Ech from a polysaccharide matrix at pH 1.3 are shown in Figure 5. About 50% of the Ech was released from the solutions of κ- and κ/β-CRG during the first hour but more that 70% from λ-CRG solution. Both the type carrageenan and the ions present are important for determining the release of Ech. The dependence of the release of Ech using sodium and potassium forms of κ-CRG is shown in Figure 5. Less than 20% of the Ech was released from the solution with the potassium form of κ-CRG. No significant differences were observed in the release profiles of Ech from the polysaccharide matrixes with different contents of CRG. With the increase in the content of CRG in the complexes (at ratios from 5:1 to 10:1 and 50:1), the amount of released Ech remained nearly constant within 2 h.

### 2.7. Mucoadhesive Properties of CRGs and Ech 

To explore the mucoadhesive properties of CRG and its mixtures with Ech, we used porcine stomach mucin, which is similar to the mucin in the epithelium of the gastrointestinal tracts of humans. Despite the popularity within the literature of determining the force of mucoadhesive attachment using rheological-based techniques, other adhesive testing methods exist that can offer alternative insights into the mechanism and degree of mucoadhesion [31,32]. 

Although polysaccharide mucoadhesion has been widely studied, the basis of these properties in CRGs remains unclear. The ability of CRGs and Ech to interact with mucin was determined by measuring their surface potential. Table 2 presents the surface potential values.

Ech has a negative charge of −30 mV. Similar to the polysaccharides in this study, mucin has a negative charge, the value of which (−13 mV) is much lower than that of CRG. The Ech-mucin mixtures had surface potentials that were close to the value of the initial mucin, which was attributed to the lack of the mucoadhesive properties of Ech. A monomodal ζ-potential distribution for the CRG-mucin mixtures was detected and the decrease in the ζ-potentials of the mixtures of all types of CRG with mucin was observed.

### 2.8. Gastroprotective Activity 

We investigated the gastroprotective effect of κ-CRG/Ech complexes in comparison with their initial components (Table 3). The samples were given to animals 1 h before indomethacin administration, which permitted the samples to protect the stomach mucosal surface from the direct contact of the ulcerogenic agent. CRG reduced the number of ulcers in the stomach by half, Ech reduced the ulcers by more than three times and κ-CRG/Ech complexes in the seven times in comparison with the control. The activity of the complexes exceeded the activity of the Phosphalugel reference drug by three times (Table 3).

## 3. Discussion

In this work, we studied the possibility of using different types of CRGs as matrixes for incorporating Ech to: improve its solubility, prevent oxidation and save or modificate its pharmacological properties. Ech is known to be water insoluble. According of our results Ech at low concentration (0.1 mg × mL^−1^) is soluble in the water solutions of all CRG-types. At the same time, CRGs decrease oxidative degradation of Ech. It should be noted that CRG with a higher degree of sulfation exhibites a stronger protective effect. Ech in the CRG solutions oxidized much more slowly than in the phosphate buffer solution, and in the solution of the phosphate buffer after 72 h, Ech was completely oxidized. 

Analysis of the differential spectra of the Ech in solutions of CRG indicates the binding between Ech and CRG. The differential spectra of Ech in water-alcohol solutions and in the 0.01 M phosphate buffer (pH 5.9), are almost identical (Figure 2b). During the transition from an aqueous-alcoholic solution, in which the Ech was present in its neutral form in a phosphate buffer solution, long wave shifts in the Ech absorption were observed from 342.5 nm to 356 nm, along with a marked decrease in its absorption. At the same time, in the range from 550 to 600 nm, broad diffuse absorption band appeared. Considering that the pH of the aqueous solution of CRG was 5.85, it is possible to assume that at least one of the three β-OH-groups of Ech was ionized [33]. The observed changes in the Ech absorption spectra in the range of 300–700 nm during the transition from the neutral to ionized state at a pH of 5.9 explain the appearance of the bands at 376 and 580 nm in the differential absorption spectra. These results indicate that Ech interacted with CRG according to an ionic mechanism, and, probably, an Ech monosodium salt was formed. CRG can also form complexes with Ech due to the intermolecular hydrogen bonds formed by the large number of hydroxyl groups present in both CRG and Ech. An increase in the concentration of CRG in the CRG/Ech solutions from 5:1 to 25:1 lead to a more than twofold increase in the absorption at the maxima of the difference bands at 376 and 580 nm (Figure 2, line a4), which is consistent with our assumption.

Various physicochemical parameters, such as the molecular size and shape, charge and molecular surface properties appear to play crucial roles in the absorption of drugs that cross the intestinal barrier through passive diffusion. The surface properties of the materials, including biopolymers that are in contact with other substances, are crucial. The comparative electron microscope examination between CRG and its mixtures with Ech showed that the CRG morphology was significantly changed after interacting with Ech. The SEM data confirmed the inclusion of Ech in the supramolecular structure of CRG and its interactions with the polysaccharide. 

The electrokinetic measurements confirm the formation of CRG/Ech complexes. A monomodal ζ-potential distribution was observed for the mixtures of CRG with Ech. The overall value of the charges of the particles in the complex was not the sum of the charges of the original components. The charge was less than that of the CRG themselves and much larger than the value of the Ech. This was a result of Ech incorporating into the CRG structure and transforming the supramolecular organization, according to the SEM data (Figure 4c,d). This is consistent with the fact that increasing the CRG content in the mixture (at ratio of CRG/Ech 40:1 *w*/*w*) resulted in the formation of unimodal charge particles (−67 mV). Two populations of particles varying in ζ-potential were observed for the mixture of λ-CRG with Ech, one of which had a low charge value (−18 mV). This result was probably due to the high degree of sulfation of λ-CRG and its macromolecular structure. 

The Ech release from the λ-CRG solution was initially quicker than that of the κ-CRG solution, which was likely related to their different macromolecular organization as what we showed earlier [34]. Our earlier studies have shown that κ-CRG and κ/β-CRG have the ability to form three-dimensional networks of ordered structures at low concentrations, whereas λ -CRG is not capable of forming ordered rigid structures [34]. This may suggest that the interactions between Ech and λ-CRG are weak, and the drug was released from its carrier once the complex was dissolved (Figure 5). The effect of potassium ions on the properties of κ-CRG is well-known. In general, adding potassium salt increased the stability of the ordered conformation and promoted gelation [35]. As a result, a slower release of Ech from potassium forms of κ-CRG was observed. It can be assumed that the Ech interacted with the polysaccharide and became embedded in the gaps of the three-dimensional network of the κ-CRG. 

The process of mucoadhesion involving a polymeric drug delivery platform is complex. The success and degree of mucoadhesion binding is influenced by various polymer-based properties, such as the degree of crosslinking, the chain length and the presence of various functional groups [32,36]. Mucosal membranes are the moist surfaces that line the walls of various body cavities, such as the gastrointestinal tracts. They consist of connective tissues covered by an epithelial layer, the surface of which is covered by mucus [37]. The major components of all mucus gels are mucin glycoproteins. The mucoadhesive properties of various substances are mainly determined by their ability to adsorb on mucin surfaces. Mucoadhesive substances that can interact with the components of the mucosa and remain in contact for a long time enable different methods for introducing active drugs [38]. 

The charge values of the particles formed by the mucin with CRG, at different component ratios, were much less than those of the CRGs, which may have been due to partial charge compensation. Mucin glycoproteins form highly entangled networks of macromolecules that associate with one another through noncovalent bonds [32,39]. The decrease in the ζ-potentials of the mixtures of all types of CRG with mucin observed at specific ratios probably resulted from the adsorption of carrageenan on the mucin surface and diffusion of the polysaccharide chains into the gaps and loops of the mucin glycoprotein mesh. With the increasing mucin content, a decrease in the charge of the CRG/mucin was observed, which agrees with the above suggestion (Table 2). For the mixture of κ-and κ/β-CRG with mucin, the greatest change in the charge was observed, which was probably due not only to the lower degree of sulfation of these polysaccharides but also to the features of their macromolecular structures. It is well accepted that the structures of polymers significantly influence their extent of diffusion, entanglement and, hence, mucoadhesion. We have previously shown that cavities exist in the macromolecular structures of CRG. In the case of κ/β-CRG, these cavities are massive [34]. The presence of these cavities probably contributed to the high swelling and binding of CRG on the mucin surface. For mucoadhesive processes, swelling is favourable because it enables greater drug release control and increases the surface area for polymer/mucus interpenetration, enhancing the mucoadhesive characteristics due to the formation of strong hydrogen bonding interactions with mucin [32]. The complexes of CRG/ECh preserved their mucoadhesive properties due to maintaining their charge (Table 2). 

Mucoadhesive polymers may offer increased intimacy with the lining of the gastrointestinal tract and, hence, the bioavailability [40]. Oral ingestion is the predominant and most preferable route for drug delivery. Previously, we found that κ-CRG possesses mild gastroprotective activity [41]. The effects of using echinochrome have not been shown previously. In the present work, we found that Ech has a moderate antiulcer effect. The further study κ-CRG, Ech and their complexes in comparison with drug Phosphalugel showed that the activity of the CRG/Ech complexes exceeded the activity of the Phosphalugel reference drug by three times. However, the complexes with different contents of CRGs had varying gastroprotective effects. The κ-CRG/Ech complex (5:1 *w*/*w*) demonstrated the most significant activity, which was attributed to the decreased quantity of Ech in of κ-CRG/Ech complex (10:1 and 20:1 *w*/*w*) (Table 3).

## 4. Materials and Methods

### 4.1. Algal Material

The following representative species of red algae were collected at the Peter the Great Bay (Sea of Japan), which is near the border between the boreal and tropical zones: Order Gigartinales, Familes: Gigartinaceae—*Chondrus armatus* (vegetative form) (*Harv*.) *Okam*; Tichocarpaceae—*Tichocarpus crinitus (Gmel) Rupr* (vegetative form) and Phyllophoraceae—*Ahnfeltiopsis flabelliformis* (reproductive form). All algae were harvested at the end of August and identified by Prof. Titlyanov E. and Titlyanova T. (National Scientific Center of Marine Biology, Russia). The algae were washed with tap water in order to remove the excess of salt. The seaweed was bleached by maintaining the specimens in pure acetone for 3 days prior being dried in the air.

### 4.2. Extraction of the Carrageenans (CRG)

The dried and milled algae (50 g) was suspended in hot water (1.5 L), and the polysaccharides were extracted at 90 °C for 2 h in a boiling water bath. The polysaccharides were separated into gelling KCl-insoluble and non-gelling KC1-soluble fractions as described previously [28], and their structures were established according to a published protocol [28,29,30].

Viscosimetric molecular weights of CRGs were calculated using the Mark-Houwink equation: [η] = *KM^α^*, where [η] is the intrinsic viscosity and *K* and α are empirical constants constituting 3 × 10^−3^ and 0.95 at 25 °C in 0.1 M NaCl for CRGs , according to the literature data for this polymer-solvent system [42].

### 4.3. The Substance Echinochrome

The sea urchin *Scaphechinus mirabilis* was collected in the Peter the Great Bay in the Sea of Japan at a depth of 5 m. Echinochrome was isolated from this sea urchin species as described in [42]. The purity of Ech (99.0%) was confirmed by melting point and LC-MS data (Shimadzu LCMS-2020, Kyoto, Japan). Purified Ech appeared like red-brown needles, was soluble in ethanol, had a m.p. of 219–221.5 °C and similar NMR spectra to that reported previously [43].

Ethanolic solution of Ech in concentration 10 mg/mL was used as a stock solution. 

### 4.4. Preparation of Ech Water Solutions with Carrageenan

Ech was added to 0.05%, 0.1% and 0.5% carrageenan water solutions to a concentration of 0.1 mg × mL^−1^. As a result, Ech water solutions with carrageenan at ratios of 5:1 and 10:1 50:1 were obtained.

### 4.5. Preparation of the Complex with Mucin

1 mL of a mucin solution (1 mg × mL^−1^ or 5 mg × mL^−1^) in water was added to 1 mL of the carrageenan solution (C = 0.5 mg × mL^−1^) in water (pH 5.9) containing Ech (0.1 mg × mL^−1^), and then, it was stored for 1 or 18 h before analysis.

### 4.6. Stability of the Ech in Water Solutions Carrageenan

The solutions of Ech with carrageenan (10 mL) were kept in glass tubes at room temperature. The absorbance of the solutions was measured at 470 nm after 0, 5, 29 and 53 h using a µQuant BioTek spectrophotometer (BioTek U.S., Winooski, VT, USA).

### 4.7. The Protective Effect of Water Solutions on Different Types of Carrageenans 

2 mL of a carrageenan solution (C = 1 mg × mL^−1^) in water (pH 5.9) was added to 0.05 mL of an ethanol solution with Ech (C = 5 mg × mL^−1^). Simultaneously, the same Ech solution was prepared in a 0.01 M phosphate buffer with a pH of 5.9. The solutions were left at room temperature in glass test tubes. After a certain period of time, the concentration of Ech in the solution was measured using absorption spectra at λ = 472 nm.

### 4.8. Differential Absorption Spectra of the Carrageenan and Ech

To register differential absorption spectra (DSP-DAS) of the carrageen solutions with Ech, solutions 1 and 2 were compared with solutions 3 and 4. Solution 1: 0.85 mL of an aqueous solution of carrageenan (C = 2 mg × mL^−1^), 0.1 mL of EtOH and 0.1 mL of an alcoholic solution of Ech (C = 2.08 mg × mL^−1^). Solution 2: 0.85 mL of water and 0.2 mL of EtOH. Solution 3: 0.85 mL of the carrageenan solution and 0.2 mL of EtOH. Solution 4: 0.85 mL of water, 0.1 mL of EtOH and 0.1 mL of an alcoholic solution of Ech (C = 2.08 mg × mL^−1^). The spectra of solutions 1 and 2 were recorded in two identical quartz cells (a layer thickness of 2 mm) against solutions 3 and 4 in the same cuvettes. Differential spectra as absorption spectrum of Ech in phosphate buffer versus water ethanol of Ech spectrum was obtained.

### 4.9. The Release of Ech from Solutions with Carrageenan 

The Ech release from solutions with carrageenan in vitro was measured using a dialysis method. Ech was dissolved in solutions of carrageenans at different ratios (5:1; 10:1 and 50:1) and placed into dialysis bags (3500 Da). The bags were then placed into 50 mL of 0.1 M hydrochloric acid solutions (pH 1.1) simulating the conditions of the human stomach [44]. The Ech release through the membrane into the acidic medium was determined under stirring with an MS01 magnetic stirrer (ELMI Ltd., Riga, Latvia) at a rotation speed of 100 rpm. After an hour, the medium was changed and Ech was extracted using 15 mL of chloroform. The chloroform was evaporated under reduced pressure, and the residue was dissolved in 10 mL of acidified ethanol (1 mL of 1 M HCl was added to 100 mL of ethanol). The release of Ech was assayed using a Shimadzu UV mini-1240 spectrophotometer (Japan) with an absorption wavelength of 470 nm. The concentration of Ech was calculated by interpolation from a calibration curve containing increasing concentrations of Ech. All experiments were performed in triplicate. 

### 4.10. Dynamic Light Scattering (DLS) and Electrophoretic Properties of the CRG-Ech Complexes

The sizes and ζ-potentials of the initial polysaccharides and their PECs in solution were determined using a ZetaSizer NanoZS system (Malvern Instruments Ltd., Worcestershire, UK) operated at 633 nm. Prior to measurements, the samples were left for 1 h to allow the large aggregates to settle because they can interfere with the measurements, even if their content does not exceed a few percent. The measurements were performed at 25 °C. The hydrodynamic diameters of the particles were automatically calculated using the software of the instrument based on analysing of the autocorrelation function. The ζ-potentials were calculated from the experimentally determined electrophoretic mobility values using the Henry equation. All measurements were done in three replicates.

### 4.11. Scanning Electron Microscopy Study

For the scanning electron microscopy studies, solution samples were placed onto the surface of Thermanox^®^ plastic coverslips (Thermo Fisher Scientific, Waltham, MA, USA), fixed with 2.5% glutaraldehyde, incubated for 1 h for sedimentation, and dehydrated in alcohols of increasing concentrations and acetone. Thereafter, the samples were completely dried in carbon dioxide according to the critical-point drying method using a BALTEC 030 (BAL- TEC AG, Balzers, Liechtenstein). The samples were then placed on the surfaces of aluminium substrates and coated with chromium. The samples were analysed using a Carl Zeiss Sigma 300 VP scanning electron microscope (Carl Zeiss Ltd., Cambridge, UK).

### 4.12. Investigation of the Gastroprotective and Antiulcerogenic Activity

The experiments were carried out using 35 Vistar female rats weighing 180–200 g in accordance with the Guidelines for the Care and Use of Laboratory Animals. Laboratory of Pharmacological Researches of Medicine Chemistry Department, N.N. Vorozhtsov Novosibirsk Institute of Organic chemistry is accredited as satisfying to the international standards ISO/IEC 17025-2009, approval code ROSS RU. 001.5104835$ N 0001912.

The animals were given standard granulated food and water ad libitum. Each group consisted of 5 animals. 

#### 4.12.1. Models of Stomach Ulcers

All animals were deprived of food with maintenance of free access to water during 24 h. Then, the rats were orally administered 20 mg × kg^−1^ indomethacin (Sigma-Aldrich, St. Louis, MO, USA) suspended in water with Tween-80 (ICN Biomedicals, Irvine, CA, USA), and 2 h later, the animals were fed. Throughout one day the rats were anaesthetized with chloroform and necropsied; their stomachs were removed, and the ulcers on the mucous membrane were counted under a binocular loupe.

The κ-CRG and Ech were diluted in water at concentrations of 0.5 mg × mL^−1^ and 0.1 mg× mL^−1^, respectively. The complexes of κ-CRG/Ech = 5:1 and 10:1 were prepared as mentioned above. Each sample was administered orally one hour before indomethacin treatment in a volume of 1 mL × 100 g^−1^ of body weight. The control group received the equivalent quantity of pure water, and the reference group received the gastroprotective drug Phosphalugel (aluminium phosphate gel, Astellas Pharma, Leiden, The Netherlands) in the same dose as κ-CRG (0.5 mg × mL^−1^ × 100 g^−1^).

For estimation, the gastroprotective effect of the samples, the Pauls indexes (PIs), were counted according to the following formula: PI = A × B/100, where A is the average number of ulcers per animal and B is the number of animals with ulcers in the group (%). The antiulcer activity (AUA) was defined as the ratio of the PI of the control group divided by the PI of the treatment group. The monitoring agent was considered to have antiulcer activity on the assumption that its AUA ˃ 2 units.

The results were analysed using Statistica 6.0 software. The differences were significant if *p* < 0.05.

#### 4.12.2. Statistical Analysis

All measurements were done in three replicates. All results were expressed as the mean ± the standard deviation (SD) and compared using ANOVA; all differences were considered to be statistically significant if *p* < 0.05. The data were analysed using the Statistica 6.0 software (StastSoft, Palo Alto, CA, USA).

## 5. Conclusions

It was shown that Ech interacted with CRGs that improved its solubility. The inclusion of Ech in complexes with CRGs decreased its oxidative degradation. The interactions caused changes in the morphology, charge and size of the polysaccharides. The CRG and its complexes with Ech exhibited mucoadhesive properties. The kinetic study of the release of Ech from CRG solutions indicated that the release rate of the active substance depends on the structure of used polysaccharide and the specific ions. Complexes of CRG/Ech exhibited significant gastroprotective activity. The activity of the complexes exceeded the activity of the reference drug Phosphalugel. New polysaccharide-based matrices may be beneficial for oral administration and prolonging the action of Ech.

## Figures and Tables

**Table 1:** Chemical structures of the repeating units of carrageenans (CRG) from algae belonging to families Gigartinaceae, Tichocarpaceae and Phyllophoraceae.

Source of Carrageenans	Type of CRG	The Disaccharide Repeating Units	Mw, kDa	Reference
*C. armatus*	Kappa (κ)	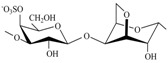	560	[28]
	Lambda (λ)	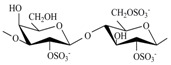	185	[28]
*A. flabelliformis*	Iota/kappa (ι/κ—70:30)	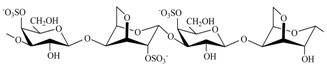	330	[30]
*T. crinitus*	Kappa/beta (κ/β—60:40)	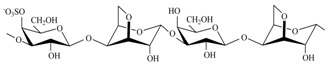	172	[29]

**Table 2:** ζ-potentials of mucin, CRGs and its complexes with Ech.

Samples	ζ-Potential, mV				
	-	Mix Carrageenan-Mucin		Mix Carrageenan-Ech-Mucin	
		1:1 *w*/*w*	1:5 *w*/*w*	1:1 *w*/*w*	1:5 *w*/*w*
Mucin, 1 mg × mL^−1^	−13.0 ± 0.2				
Ech, 0.1 mg × mL^−1^	−30.4 ± 1.1				
Ech/mucin (1:10)	−14.1 ± 2.0				
κ-CRG, 0.5 mg × mL^−1^	−70.1 ± 1.1	−50.8 ± 1.0	−34.8 ± 0.5	−44.6 ± 2.4	−51.5 ± 0.8
κ/β-CRG, 0.5 mg × mL^−1^	−68.6 ± 3.0	−44.0 ± 1.5	−45.5 ± 1.7	−38.6 ± 0.4	−31.2 ± 0.6
κ/ι-CRG, 0.5 mg × mL^−1^	−61.2 ± 1.3	−51.7 ± 0.6	−30.6 ± 0.5	−53.2 ± 0.4	−29.1 ± 0.3
λ-CRG , 0.5 mg × mL^−1^	−66.6 ± 3.7	−50.7 ± 1.1	−46.6 ± 0.7	−45.6 ± 5.6	−33.3 ± 0.3

**Table 3:** Comparative gastroprotective activity of CRG, Ech and theirs complex in rats with stomach ulcers induced by indomethacin.

	Total Number of Ulcers in the Group, 5 Rats	The Average Number of Ulcers per 1 Rat	Pauls Index	Antiulcer Activity, Units
Control	37	7.40 ± 1.29	7.4	-
κ-CRG, 0.5 mg × mL^−1^	24	4.80 ± 1.20	4.8	1.54
Ech, 0.1 mg × mL^−1^	10	2.00 ± 1.05 *p* = 0.011	1.6	4.63
κ-CRG/Ech, (5:1 *w*/*w*)	5	1.00 ± 0.45 *p* = 0.0015	0.6	12.33
κ-CRG/Ech, (10:1 *w*/*w*)	20	4.00 ± 1.30	3.2	2.31
κ-CRG/Ech, (20:1 *w*/*w*)	22	4.40 ± 1.69	3.5	2.11
Phosphalugel, 0.5 mg × mL^−1^	10	2.00 ± 0.71 *p* = 0.0062	1.6	4.63

*p* ≤ 0.05—differences with control are significant.

## References

[1] Molinski T.F., Dalisay D.S., Lievens S.L., Saludes J.P. (2009). Drug development from marine natural products. Nat. Rev. Drug Discov..

[2] Laurienzo P. (2010). Marine polysaccharides in pharmaceutical applications: An Overview. Mar. Drugs.

[3] Cardoso M.J., Costa R.R., Mano J.F. (2016). Marine Origin Polysaccharides in Drug Delivery Systems. Mar. Drugs.

[4] Knutsen S.H., Myslabodsky D.E., Larsen B., Usov A.I. (1994). A modified system of nomenclature for red algae galactans. Bot. Mar..

[5] Falshaw R., Furneaux R.H. (1994). Carrageenan from tetrasporic stage of *Gigartina decipiens.* (Gigartinaceae, Rhodophyta). Carbohydr. Res..

[6] Yermak I.M., Khotimchenko Y.S., Fingerman M., Nagabhushanam R. (2003). Chemical properties, biological activities and applications of carrageenan from red algae. Recent Advances in Marine Biotechnology.

[7] Ghosh T., Chattopadhyay K., Marschall M., Karmakar P., Mandal P., Ray B. (2009). Focus on antivirally active sulfated polysaccharides: From structure-activity analysis to clinical evaluation. Glycobiology.

[8] Pereira M.G., Benevides N.M.B., Melo M.R.S., Valente A.P., Melo F.R., Mourao P.A.S. (2005). Structure and anticoagulant activity of sulfated galactan from the red alga, *Gelidium crinale*. Is there a specific structural requirement for the anticoagulant action?. Carbohydr. Res..

[9] Zhou G., Sun Y., Xin H., Zhang Y., Li Z., Xu Z. (2004). In vivo antitumor and immunomodulation activities of different molecular weight lambda-carrageenans from *Chondrus ocellatus*. Pharmacol. Res..

[10] Bhattacharyya S., Liu H., Zhang Z., Jam M., Dudeja P.K., Michel G., Linhardt R.J., Tobacman J.K. (2010). Carrageenan-induced innate immune response is modified by enzymes that hydrolyze distinct galactosidic bonds. J. Nutr. Biochem..

[11] Yermak I.M., Barabanova A.O., Aminin D.L., Davydova B.N., Sokolova E.V., Yong H.K., Kwang S.S., Solov’eva T.F. (2012). Effects of structural peculiarities of carrageenans on immunomodulation and anti-coagulant activities. Carbohydr. Polym..

[12] Yermak I.M., Sokolova E.V., Davydova V.N., Solov’eva T.F., Aminin D.L., Reunov A.V., Lapshina L.A. (2016). Influence of red algal polysaccharides on biological activities and supramolecular structure of bacterial lipopolysaccharide. J. Appl. Phycol..

[13] Solov’eva T., Davydova V., Krasikova I., Yermak I. (2013). Marine Compounds with Therapeutic Potential in Gram-Negative Sepsis. Mar. Drugs.

[14] Campo V.L., Kawano D.F., da Silva D.B., Carvalho D.I. (2009). Carrageenans: Biological properties, chemical modifications and structural analysis—A review. Carbohydr. Polym..

[15] Prajapati V.D., Maheriya P.M., Jani G.K., Solanki H.K. (2014). Carrageenan: A natural seaweed polysaccharide and its applications. Carbohydr. Polym..

[16] Keppeler S., Ellis A., Jacquier J. (2009). Cross-linked carrageenan beads for controlled release delivery systems. Carbohydr. Polym..

[17] Silva T.H., Alves A., Popa E.G., Reys L.L., Gomes M.E., Sousa R.A., Silva S.S., Mano J.F., Reis R.L. (2012). Marine algae sulfated polysaccharides for tissue engineering and drug delivery approaches. Biomatter.

[18] Mohamadnia Z., Zohuriaan-Mehr A.J., Kabiri K., Jamshidi A., Mobedi H. (2007). pH-sensitive IPN hydrogel beads of carrageenan-alginate for controlled drug delivery. J. Bioact. Compat. Polym..

[19] Leong K.H., Chung L.V., Noordin M.I., Mohama K., Nichikawa M., Onuki Y. (2011). Carboxymethylation of kappa-carrageenan for intestinal-targeted delivery of bioactive macromolecules. Carbohydr. Polym..

[20] Daniel-da-Silva A.L., Moreira J., Neto R., Estrada A.C., Gil A.M., Trindade T. (2012). Impact of magnetic nanofillers in the swelling and release properties of κ-carrageenan hydrogel nanocomposites. Carbohydr. Polym..

[21] Maderuelo C., Zarzuelo A., Lanao J.M. (2011). Critical factors in the release of drugs from sustained release hydrophilic matrices. J. Control. Release.

[22] Anderson H.A., Mathieson J.W., Thomson R.H. (1969). Distribution of spinochrome pigments in echinoids. Comp. Biochem. Physiol..

[23] Elyakov G.B., Maximov O.B., Mischenko N.P., Koltsova E.A., Fedoreev S.A., Glebko L.I., Krasovskaya N.P., Artjukov A.A. (2001). Histochrome and Its Therapeutic Use in Acute Myocardial Infarction and Ischemic Heart Disease. U.S. Patent.

[24] Elyakov G.B., Maximov O.B., Mischenko N.P., Koltsova E.A., Fedoreev S.A., Glebko L.I., Krasovskaya N.P., Artjukov A.A. (2004). Composition Comprising di-and Trisodium Salts of Echinochrome for Treating Ocular Conditions. European Patent.

[25] Elyakov G.B., Maximov O.B., Mischenko N.P., Koltsova E.A., Fedoreev S.A., Glebko L.I., Krasovskaya N.P., Artjukov A.A. (2007). Drug Preparation “Histochrome” for Treating Acute Myocardial Infarction and Ischaemic Heart Diseases. European Patent.

[26] Jeong S.H., Kim H.K., Song I.-S., Noh S.J., Marquez J., Ko K.S., Rhee B.D., Kim N., Mishchenko N.P., Fedoreyev S.A. (2014). Echinochrome A increases mitochondrial mass and function by modulating mitochondrial biogenesis regulatory genes. Mar. Drugs.

[27] Seo D.Y., McGregor R.A., Noh S.J., Choi S.J., Mishchenko N.P., Fedoreyev S.A., Stonik V.A., Han J. (2015). Echinochrome A Improves Exercise Capacity during Short-Term Endurance Training in Rats. Mar. Drugs.

[28] Yermak I.M., Kim Y.H., Titlyanov E.A., Isakov V.V., Solov’eva T.F. (1999). Chemical structure and gel properties of carrageenan from algae belonging to the Gigartinaceae and Tichocapaceae, collected from the Russian Pacific coast. J. Appl. Phycol..

[29] Barabanova A.O., Yermak I.M., Glazunov V.P., Isakov V.V., Titlyanov E.A., Solov’eva T.F. (2005). Comparative study of carrageenan from reproductive and sterile forms of *Tichocarpus crinitus* (Gmel.) Rupr. (Rhodophyta, Tichocarpaceae). Biochemistry.

[30] Kravchenko A.O., Anastyuk S.D., Sokolova E.V., Isakov V.V., Glazunov V.P., Helbert W., Yermak I.M. (2016). Structural features and cytokine-induced activity of polysaccharide from reproductive form of *Ahnfeltiopsis flabelliformis*. Carbohydr. Polym..

[31] Harding S.E. (2006). Trends in mucoadhesive analysis. Trends Food Sci. Technol..

[32] Andrews G.P., Laverty T.P., Jones D.S. (2009). Mucoadhesive polymeric platforms for controlled drug delivery. Eur. J. Pharm. Biopharm..

[33] Petrova S.A., Ksenzhek O.S., Kolodyazhny M.V. (1995). Redox properties of echinochrome A. J. Electroanal. Chem..

[34] Sokolova E.V., Chusovitin E.A., Barabanova A.O., Balagan S.A., Galkin N.G., Yermak I.M. (2013). Atomic force microscopy imaging of carrageenans from red algae of Gigartinaceae and Tichocarpaceae families. Carbohydr. Polym..

[35] Rochas C., Rinaudo M. (1984). Mechanism of gel formation in K- carrageenan. Biopolymeres.

[36] Sogis I.A., Williams A.C., Khutoryanskiy V.V. (2008). Why is Chitosan Mucoadhesive?. Biomacromolecules.

[37] Jacobs C., Kayser O., Müller R.H. (2001). Production and characterisation of mucoadhesive nanosuspensions for the formulation of bupravaquone. Int. J. Pharm..

[38] Bernkop-Schnürch A. (2005). Mucoadhesive systems in oral drug delivery. Drug Discov. Today.

[39] Smart J.D. (2005). The basics and underlying mechanisms of mucoadhesion. Adv. Drug Deliv. Rev..

[40] Khutoryanskiy V.V. (2011). Advances in mucoadhesion and mucoadhesive polymers. Macromol. Biosci..

[41] Volod’ko A.V., Davydova V.N., Chusovitin E.V., Sorokina I.V., Dolgik M.P., Tolstikova T.G., Balagan S.A., Galkin N.G., Yerma I.M. (2014). Soluble chitosan–carrageenan polyelectrolyte complexes and their gastroprotective activity. Carbohydr. Polym..

[42] Rochas C., Rinaudo M., Landry S. (1990). Role of the molecular weight on the mechanical properties of kappa carrageenan gels. Carbohydr. Polym..

[43] Mischenko N.P., Fedoreyev S.A., Pokhilo N.D., Anufriev V.P., Denisenko V.A., Glazunov V.P. (2005). Echinamines A and B, First Aminated Hydroxynaphthazarins from the Sea Urchin Scaphechinus mirabilis. J. Nat. Prod..

[44] Vasileva E.A., Mishchenko N.P., Tran V.T.T., Vo H.M.N., Bui L.M., Denisenko V.A., Fedoreyev S.A. (2017). Quinoid pigments from the sea urchin *Astropyga radiata*. Chem. Nat. Compd..

